# Pharmacokinetic Profile and Anti-Adhesive Effect of Oxaliplatin-PLGA Microparticle-Loaded Hydrogels in Rats for Colorectal Cancer Treatment

**DOI:** 10.3390/pharmaceutics11080392

**Published:** 2019-08-05

**Authors:** Sharif Md Abuzar, Jun-Hyun Ahn, Kyung Su Park, Eun Jung Park, Seung Hyuk Baik, Sung-Joo Hwang

**Affiliations:** 1 College of Pharmacy, Yonsei University, 85 Songdogwahak-ro, Yeonsu-gu, Incheon 21983, Korea; 2 Yonsei Institute of Pharmaceutical Sciences, Yonsei University, 85 Songdogwahak-ro, Yeonsu-gu, Incheon 21983, Korea; 3 Advanced Analysis Center, Korea Institute of Science and Technology, Hwarang-ro, Seongbuk-gu, Seoul 02792, Korea; 4 Division of Colon and Rectal Surgery, Department of Surgery, Gangnam Severance Hospital, Yonsei University College of Medicine, Seoul 06273, Korea

**Keywords:** oxaliplatin, PLGA, hydrogel, intra-abdominal anti-adhesion barrier, colorectal cancer

## Abstract

Colorectal cancer (CRC) is one of the most malignant and fatal cancers worldwide. Although cytoreductive surgery combined with chemotherapy is considered a promising therapy, peritoneal adhesion causes further complications after surgery. In this study, oxaliplatin-loaded Poly-(d,l-lactide-co-glycolide) (PLGA) microparticles were prepared using a double emulsion method and loaded into hyaluronic acid (HA)- and carboxymethyl cellulose sodium (CMCNa)-based cross-linked (HC) hydrogels. From characterization and evaluation study PLGA microparticles showed smaller particle size with higher entrapment efficiency, approximately 1100.4 ± 257.7 nm and 77.9 ± 2.8%, respectively. In addition, microparticle-loaded hydrogels showed more sustained drug release compared to the unloaded microparticles. Moreover, in an in vivo pharmacokinetic study after intraperitoneal administration in rats, a significant improvement in the bioavailability and the mean residence time of the microparticle-loaded hydrogels was observed. In HC21 hydrogels, AUC_0–48h_, C_max_, and T_max_ were 16012.12 ± 188.75 ng·h/mL, 528.75 ± 144.50 ng/mL, and 1.5 h, respectively. Furthermore, experimental observation revealed that the hydrogel samples effectively protected injured tissues from peritoneal adhesion. Therefore, the results of the current pharmacokinetic study together with our previous report of the in vivo anti-adhesion efficacy of HC hydrogels demonstrated that the PLGA microparticle-loaded hydrogels offer novel therapeutic strategy for CRC treatment.

## 1. Introduction

Colorectal cancer (CRC) is one of the most malignant cancers worldwide. Although the incidence of CRC is low, the number of new cases that are malignant and fatal is still the highest among both men and women. In addition, peritoneal carcinomatosis is one of the numerous manifestations of CRC identified at the first diagnosis in more than 10% of CRC patients, and it is extremely fatal, with median survival of approximately 6 months [1,2]. The treatment strategies for both CRC and peritoneal carcinomatosis patients depend on the type and stage, and optimal therapy comprises cytoreductive surgery combined with chemotherapy [3].

Peritoneal adhesion is one of the most common postoperative complications associated with cytoreductive surgery. Previous studies indicated their occurrence in more than 50% cases, and they have even higher recurrence rates (85–93%) [4,5]. As cytoreductive surgery and intraperitoneal chemotherapy are closely related to the completeness of cytoreduction, problems associated with cytoreductive surgery are still a big challenge in medical science. Therefore, hydrogel-based anti-adhesion barriers came into the spotlight to reduce adhesion by mechanical separation of injured tissue surfaces during peritoneal repair after surgery [6].

**Figure 1:**
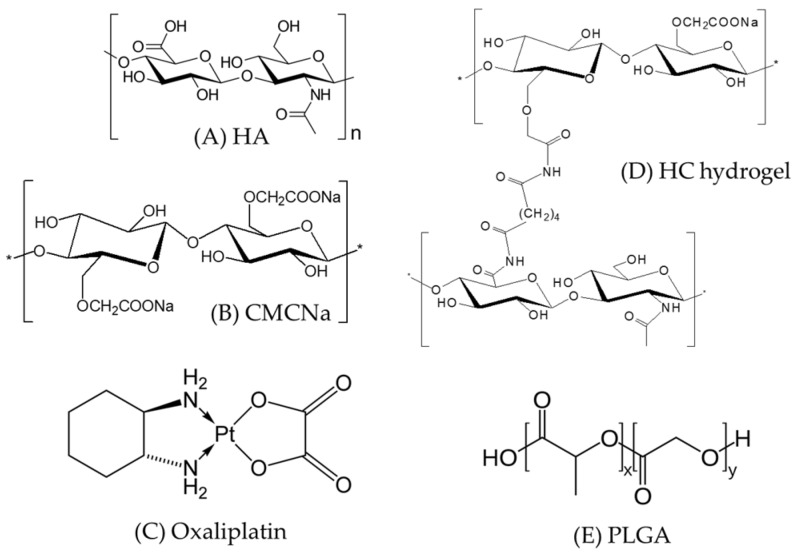
Chemical structures of Poly-(d,l-lactide-*co*-glycolide) (PLGA) microparticles and hyaluronic acid (HA) and carboxymethyl cellulose sodium (CMCNa)-based cross-linked (HC) hydrogel components (**A**) HA; (**B**) CMCNa; (**C**) HC hydrogel; (**D**) Oxaliplatin; and (**E**) PLGA.

Oxaliplatin (Figure 1) is a third-generation, platinum-based, systemic chemotherapeutic agent for CRC [7], and it is expected to be similarly effective in peritoneal carcinomatosis. Therefore, it is currently being used as a part of the standard chemotherapy regimen, FOLFOX (oxaliplatin with 5-fluorouracil and leucovorin) [8], for the clinical treatment of metastatic CRC [9]. However, a recent study conducted in a murine model confirmed that intraperitoneally administered oxaliplatin enhanced peritoneal tissue concentration while reducing its systemic absorption, suggesting a possible decrease in toxicity associated with systemic chemotherapy [10]. Therefore, the new approach comprising cytoreductive surgery followed by intraperitoneal oxaliplatin delivery has resulted in significant improvement in the disease states of CRC and peritoneal carcinomatosis [11]. 

Biodegradable microparticles have been widely investigated for the controlled delivery of chemotherapeutic agents [12,13]. In addition, neither an initial burst release, nor the presence of a lag phase are desirable for the chemotherapeutic agents as it can be associated with adverse effects. Poly-(d,l-lactide-*co*-glycolide) (PLGA) is an unique copolymer approved by the regulatory agencies for the manufacturing of bioresorbable surgical sutures. Owing to its biodegradability and biocompatibility, PLGA become the first line materials for the production of injectable microparticle based controlled release system [14]. A recent report of metformin/irinotecan-loaded nanoparticles have shown an initial 2 h burst release, however, increase the drug retention time in tumor and increase drug circulation [15]. Moreover, alternative reports of paclitaxel-loaded PLGA microparticles have been evaluated for cancer therapy. These models revealed protection of the therapeutic payload from premature burst release, and enable the sustained release of paclitaxel [16,17].

Recently we succeeded in synthesizing novel hyaluronic acid (HA) and carboxymethyl cellulose sodium (CMCNa)-based cross-linked (HC) hydrogels loaded with oxaliplatin [18]. These novel HC hydrogels significantly prevented intraperitoneal adhesion and offered the highest anti-adhesion barrier in an in vivo rat model. However, pharmacokinetic evaluation of oxaliplatin-loaded HC hydrogels was not carried out. Several reports of microparticles-loaded hydrogels in various drug delivery system showed controlled drug release for over a prolonged period [19,20]. Therefore, to provide an adequate anti-adhesion barrier effect after cytoreductive surgery and deliver intraperitoneal chemotherapy, oxaliplatin-loaded PLGA microparticles were prepared, characterized, and loaded into HC hydrogels. In addition, an in vitro oxaliplatin release study of PLGA microparticle-loaded HC hydrogels in comparison with that of commercially available Guardix-Sol^®^ hydrogel was carried out. Furthermore, in vivo pharmacokinetic analysis was carried out to demonstrate the efficacy of intraperitoneal chemotherapy with oxaliplatin-PLGA microparticles loaded into HC hydrogels.

## 2. Materials and Methods

### 2.1. Materials and Animals

Oxaliplatin was a gift from Boryung Pharm (Ansan, Korea). Resomer^®^ RG 502 H (Poly-(d,l-lactide-*co*-glycolide)) PLGA, molecular weight (MW) approximately 7000–17,000, was purchased from Evonic Ind., (Darmstadt, Germany). Hyaluronic acid (HA, MW 1000 kDa) was a gift from Huons (Seongnam, Korea). Poly-(vinyl alcohol) (PVA, MW approximately 89,000–98,000), carboxymethyl cellulose sodium (CMCNa, MW approximately 700 kDa), and adipic acid dihydrazide (ADH) were obtained from Sigma Aldrich (St. Louis, MO, USA). 1-Ethyl-3-(3-(dimethylaminopropyl) carbodiimide (EDC) was purchased from Tokyo Chemical (Tokyo, Japan). All other chemicals were of reagent grade, and Milli-Q^®^ water (Millipore1, Molsheim, France) was used throughout the study.

Male Sprague-Dawley (SD) rats were purchased from YoungBio (Seongnam, Republic of Korea). The animals were housed in a semi-specific pathogen free facility using standard cages at 19 ± 1 °C and 50 ± 5% relative humidity, with a 12-h light-dark cycle. The rats were fed a standard diet, provided with purified water, and allowed to move freely. All experiments were approved by the Institutional Animal Care and Use Committee (IACUC-201809-788-01) at Yonsei University, Seoul, Korea, and were performed according to IACUC guidelines. 

### 2.2. Preparation of Oxaliplatin-PLGA Microparticles

Oxaliplatin-PLGA microparticles were prepared using a double emulsion method [21]. Briefly, oxaliplatin (20 mg) was dissolved in 5 mL of deionized water (first aqueous solution, W_1_) containing 0.5% (*w*/*v*) PVA. The resulting oxaliplatin containing W_1_ was added dropwise to 12.5 mL DCM, containing 1 g of PLGA 502 H, using a homogenizer ULTRA-TURRAX^®^ (IKA-WERKE GMBH & Co. KG, Staufen, Germany) at 12,000 rpm for 2 min. This primary emulsion (W_1_/O) was slowly added to 100 mL of 1% (*w*/*v*) PVA solution (second aqueous solution, W_2_) and emulsified at 400 rpm using magnetic stirrer for 3 h while the DCM was allowed to evaporate completely under vacuum. The resultant microparticles were collected, washed using distilled water, centrifuged, and freeze-dried using an ilShinBioBase (Seoul, Korea) freeze-drier under vacuum (5 mTorr). Samples were pre-frozen for 2 h at −60 ± 1.0 °C prior to final drying at −88 ± 1.0 °C for 2 days. The collected freeze-dried microparticles were stored at 4 °C and further evaluated by scanning electronic microscopy (SEM) to characterize the morphological features.

### 2.3. Synthesis of HA-CMCNa Cross-Linked Hydrogels

Hydrogels were synthesized using variable ratios of HA and CMCNa (Figure 1) by chemical cross-linking, as described by our previous study [18]. Briefly, HA and CMCNa at ratios of 1:2 and 2:1 (*w*/*w*) were dissolved in deionized water with continuous stirring. After complete dissolution, 2 mM/L of ADH was added to the mixture, and the pH was adjusted to 4.75 ± 0.05 using 0.1 M HCl. After vortex mixing for 1 min, 2 mM/L EDC was added, and the resulting mixture was stirred thoroughly at room temperature (25 ± 1.0 °C) for at least 12 h to allow for complete cross-linking. The reaction was maintained at pH 4.75 ± 0.05 by the addition of 0.1 M HCl. Finally, the cross-linking reaction was terminated by elevating the pH of the mixture to 7.0 ± 0.05 via the slow addition of 0.1 M NaOH. The hydrogel samples were purified as previously described by Luo et al. [22]. Briefly, the hydrogel samples were dispensed into a dialysis bag (Mw cut-off 18,000 Da) previously treated with a 70% ethanol (EtOH) solution and dried. The bag was submerged and dialyzed for 12 h in 0.1 M NaCl followed by alternating solutions of 25% EtOH for 6 h and DW for 6 h. The remaining hydrogels in the dialysis bag were rinsed using EtOH, and then centrifuged at 2000 rpm for 10 min to remove the remaining ADH. Finally, the precipitated hydrogels (HC12 and HC21) were collected and stored until further experiments.

### 2.4. Preparation of Oxaliplatin-PLGA Microparticle-Loaded Hydrogel

Guardix-Sol^®^ is a commercial anti-adhesive hydrogel used for the prevention of post-operative adhesion. Above, we synthesized HC12 and HC21 hydrogel composites with variable weight ratios of HA and CMCNa. Next, oxaliplatin-PLGA microparticles were dispersed gently in the hydrogel (HC12, HC21, and Guardix-Sol^®^). Briefly, precisely weighed oxaliplatin-PLGA microparticles were mixed with 10 mL of hydrogels at room temperature (25 ± 1.0 °C) with continuous stirring for 30 min. The final concentration of oxaliplatin was 2 mg/ mL of hydrogel. After complete dissolution was visually observed, samples were evaluated for in vitro and in vivo release of oxaliplatin.

### 2.5. Characterization of Oxaliplatin-PLGA Microparticles

#### 2.5.1. Morphology

The morphology of oxaliplatin-PLGA microparticles was evaluated using SEM (JSM-6700F, JEOL, Tokyo, Japan). Briefly, a small amount of powder was sprinkled onto double-sided adhesive tape attached to an aluminum stub and was sputter-coated with gold under vacuum. Photographs were taken at 5× magnification with an accelerating voltage of 1–5 kV to reveal the surface characteristics of the particles.

#### 2.5.2. Particle Size Analysis

The mean particle size and distribution of oxaliplatin-PLGA microparticles were analyzed by dynamic light scattering (DLS) using an electrophoretic light scattering spectrophotometer (ELS-Z, Otsuka Electronics, Hirakata, Japan).

#### 2.5.3. Rheological Measurements

The rheological measurements for HC12, HC21, and Guardix-Sol^®^ hydrogels were performed using a Brookfield rheometer (Brookfield Digital Rheometer Model DV-III, DV3T™ Rheometer, Middleboro, MA, USA) equipped with a Peltier system for temperature control. Precisely, about 0.5 g of sample was applied to the plate and allowed to equilibrate. Measurements were performed at 37 ± 0.5 °C with shear rates ranging from 200–500 s^−1^. Before each measurement, the samples were allowed to rest for 5 min at 37 ± 0.5 °C. Results were analyzed with Brookfield software (Firmware version 1.2.2-9).

#### 2.5.4. Encapsulation Efficiency

Freeze-dried oxaliplatin-PLGA microparticles (eq. 2 mg of oxaliplatin) were dissolved in 5 mL of DCM. After 5 min of vortexing, particles were allowed to dissolve properly, and the tube was gently swung in incubator at 37 ± 0.5 °C for 1 h. Then, 5 mL of DW was added to the tube and vortexed vigorously for 1 h. The suspension was centrifuged at 12,000 rpm for 5 min to precipitate PLGA. The upper aqueous phase was collected, and the concentration of oxaliplatin was determined by HPLC. Encapsulation efficiency (EE %) was calculated using the following Equation (1):

EE % = M_actual oxaliplatin_/M_theoretical oxaliplatin_ × 100 (1)

### 2.6. In Vitro Oxaliplatin Release from PLGA Microparticles Loaded into Hydrogels

To evaluate the in vitro oxaliplatin release rate from oxaliplatin-PLGA microparticles, an amount equivalent to 2 mg of oxaliplatin was weighed and suspended in 1 mL of HPLC grade water. The suspension was transferred into dialysis bags (MWCO 14,000) and then submerged in 20 mL of double distilled water in capped 50 mL Falcon^®^ tube. Samples were shaken at 30 rpm at the predetermined time points (1, 2, 3, 4, 6, 8, and 12 h); 1 mL of sample was collected from the medium and 1 mL of pre-warmed medium was immediately added to the tubes. Experiments were performed in triplicate (n = 3), and the collected samples were analyzed with HPLC after necessary dilution. The release profile was expressed as the ratio of cumulative oxaliplatin release to initial oxaliplatin loading versus time, using the following Equation (2):

Percent of oxaliplatin release = M_t_/M_0_ × 100 (2)

For the in vitro oxaliplatin release from the PLGA microparticle-loaded hydrogels (from Section 2.4.), a similar method was used. Briefly, 1 mL PLGA microparticle-loaded hydrogel (HC12, HC21, or Guardix-Sol^®^) was transferred to one end-closed dialysis bags (MWCO 14,000). The other end of the bag was closed properly, and the samples were submerged in 20 mL of double distilled water in capped 50 mL Falcon^®^ tube. Samples were shaken at 30 rpm at similar time points (1, 2, 3, 4, 6, 8, and 12 h). Subsequently, 1 mL of sample was collected from the medium and 1 mL of pre-warmed medium was immediately added to the tubes. Experiments were performed in triplicate (n = 3), and the collected samples were analyzed with HPLC after necessary dilution with the mobile phase. The release profile was expressed as cumulative oxaliplatin release calculated using Equation (2).

### 2.7. In Vivo Oxaliplatin Release in SD Rat’s Intraperitoneal Cavity

In vivo oxaliplatin release from the prepared hydrogels was evaluated in the intraperitoneal cavity of SD rats, and oxaliplatin solution was also evaluated for comparison. Briefly, a total of fifteen male SD rats, aged 4–6 weeks, were randomly divided into three groups (n = 5, per group) and were anesthetized with isoflurane. Intra-abdominal adhesions were induced to mimic the postoperative surgical conditions, as described previously [23]. The peritoneum was exposed by a 5-cm ventral midline incision. The left abdominal sidewall was scraped with a 1 cm^2^ piece of 100-grit sandpaper 200 times. In group 1, oxaliplatin solution was introduced, whereas, group 2 and 3 were exposed to oxaliplatin powder and oxaliplatin-PLGA microparticles, loaded into HC21 hydrogel, respectively. All the rats from each group were administered oxaliplatin in the form of a solution or loaded into hydrogel at a dose level of 5 mg/kg body weight [24]. The abdominal layers and skin incision were then completely closed, and each rat was kept separately in single cases. All surgeries were performed by the same individual. At predetermined time interval (0.5, 1, 1.5, 2, 3, 5, 8, 14, 24, and 48 h), approximately 1-mL blood samples were collected from the conjunctiva using a capillary tube and kept in an ice bath. 

Blood samples collected from the rat’s conjunctiva were centrifuged at 10,000 rpm (9425× *g*) for 10 min at 4 °C. The supernatant plasma was obtained and stored at −80 °C until analyzed. The frozen plasma samples were thawed, and approximately 200 ± 5 mg was weighed. The samples were oxidized into metals and organic materials by treating with 6 mL nitric acid using a microwave sample pre-treatment machine equipped with platinum (Pt) sensor (Microwave Reaction System, Multi-wave PRO, Anton Paar, Graz, Austria). Samples were heated at predefined temperature and pressure (200 ± 0.5 °C and 4.0 MPa) for 1 h and diluted up to 30 mL with deionized water, and the concentrations of Pt were analyzed using ICP-MS (NexION 300 D, PerkinElmer, Waltham, MA, USA). Pharmacokinetic parameters AUC_0–48h_ (area under curve), C_max_ (peak concentration), and T_max_ (time to peak concentration) were calculated using noncompartmental analysis.

After the last blood sample collection at 48 h, rats were housed individually in standard case with a 12-h light-dark cycle. The rats were fed a standard diet, provided with purified water, and were allowed to move freely. After 10 days, they were sacrificed, and the peritoneum was opened. For all rats, adhesion type and extent were assessed, and photographs were taken.

### 2.8. High-Performance Liquid Chromatography (HPLC) Analysis

In vitro oxaliplatin release and % EE were determined by using HPLC Agilent 1200 Infinity Series HPLC system (Agilent Technologies, Waldbronn, Germany). Briefly, XTerra™ RPC_18_ column (particle size 5 μm, inside diameter 4.6 mm, and length 250 mm; Waters Corporation, Milford, MA, USA) at 210 nm using an HPLC-UV spectrometer (Agilent 1290 infinity). The mobile phase was a 20:80 mixture of ACN and deionized water and was set to a flow rate of 0.8 mL/min (Model 1260 Quat Pump VL). The samples were diluted as necessary, and 20 μL of each sample was injected using an autosampler (Model 1260 ALS).2.9. Statistical Analysis

In vitro percent (%) cumulative oxaliplatin release and in vivo pharmacokinetic study data are expressed as the means ± standard deviations. The *t*-test or two-sided RM ANOVA and Bonferroni test were applied to the analyses of the differences between the groups. A *p* value < 0.05 was considered as a statistically significant difference.

## 3. Results and Discussion

### 3.1. Characterization of PLGA Microparticles

**Figure 2:**
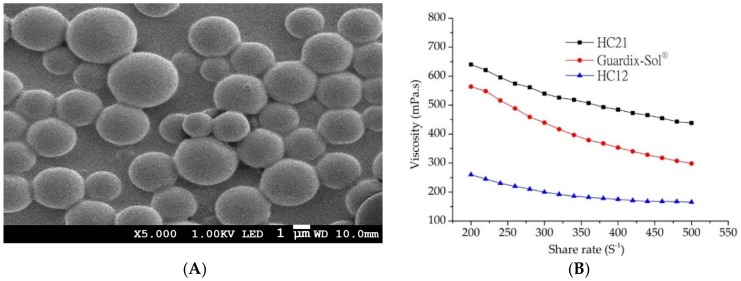
(**A**) SEM image of oxaliplatin-PLGA microparticles (at X5.000 magnification); (B) rheology properties of hydrogel samples (■) HC21, (●) Guardix-Sol^®^, and (▲) HC12 (measurements were performed at 37 ± 0.5 °C).

PLGA-based microparticles were prepared using the double emulsion method. The microparticles were characterized for size, morphology, and encapsulation efficiency. As shown in Table 1, oxaliplatin-loaded PLGA microparticles had a particle size of more than 1 µm diameter, with a small standard deviation. Encapsulation efficiency was 77.9 ± 2.8%, which is excellent for a hydrophilic drug like oxaliplatin. Although the encapsulation of hydrophilic drugs in PLGA microparticles is challenging due to the partitioning of weakly associated drugs from the oil phases to the external water phase, the double emulsion technique is the most commonly used method for encapsulating hydrophilic drugs. In addition, the SEM image shown in Figure 2A reveals the morphology of the microparticles. Uniform size and spherical particles were observed, which is in agreement with the particle size results. To fulfill the aims of this study, PLGA microparticles were further loaded into HC hydrogels and the in vitro release and in vivo pharmacokinetic studies were conducted. 

### 3.2. Preparation and Characterization of HC Hydrogels

Cross-linked HC hydrogels were prepared using variable weight ratios of HA and CMCNa. HC hydrogel synthesis was carried out based on our previous description [18]. In the presence of ADH (as a nucleophile), EDC (a water-soluble carbodiimide) linked both HA and CMCNa molecules with its amine group. EDC is not incorporated into the final product, but converts into a non-toxic water-soluble urea derivative, which is removed by dialysis. Moreover, CH_2_COO−anions provided by CMCNa react with H^+^ ions, leading to carboxyl group formation and facilitation of cross-linking. Therefore, the reaction in the presence of EDC is pH-dependent and was performed at pH 4.75 ± 0.05. To evaluate the rheology of the HC hydrogel along with the commercial product Guardix-Sol^®^, a Brookfield Digital Rheometer was used. All measurements were performed at 37 ± 0.5 °C, with shear rates ranging from 200 to 500 s^−1^. All hydrogels followed non-Newtonian shear thinning (pseudo) plastic flow behavior (Figure 2B). Decreasing viscosity with increasing shear rates was observed, which demonstrated viscosity-dependent shear rates [25]. 

In addition, variable ratios of the composition (HA and CMCNa) alter the viscosity of the HC hydrogels. The order could be written as follows: HC21 > HC12, as markedly increased viscosity was observed with HA. The highest viscosity was observed with the HC21 formulation, whereas the lowest viscosity was observed with HC12. The viscosity of Guardix-Sol^®^ was measured for comparison with those of the synthesized HC hydrogels, and it showed moderate viscosity. The rheology results correlated with the mechanical characteristics of the polymer, in which viscosity scaled linearly with molecular weight. The mechanical properties (entanglement phenomenon) of the polymers increased with increasing molecular weight. Finally, the hydrogels were loaded with oxaliplatin-PLGA microparticles and their in vitro and in vivo characteristics were evaluated.

### 3.3. In Vitro Oxaliplatin Release Study

**Figure 3:**
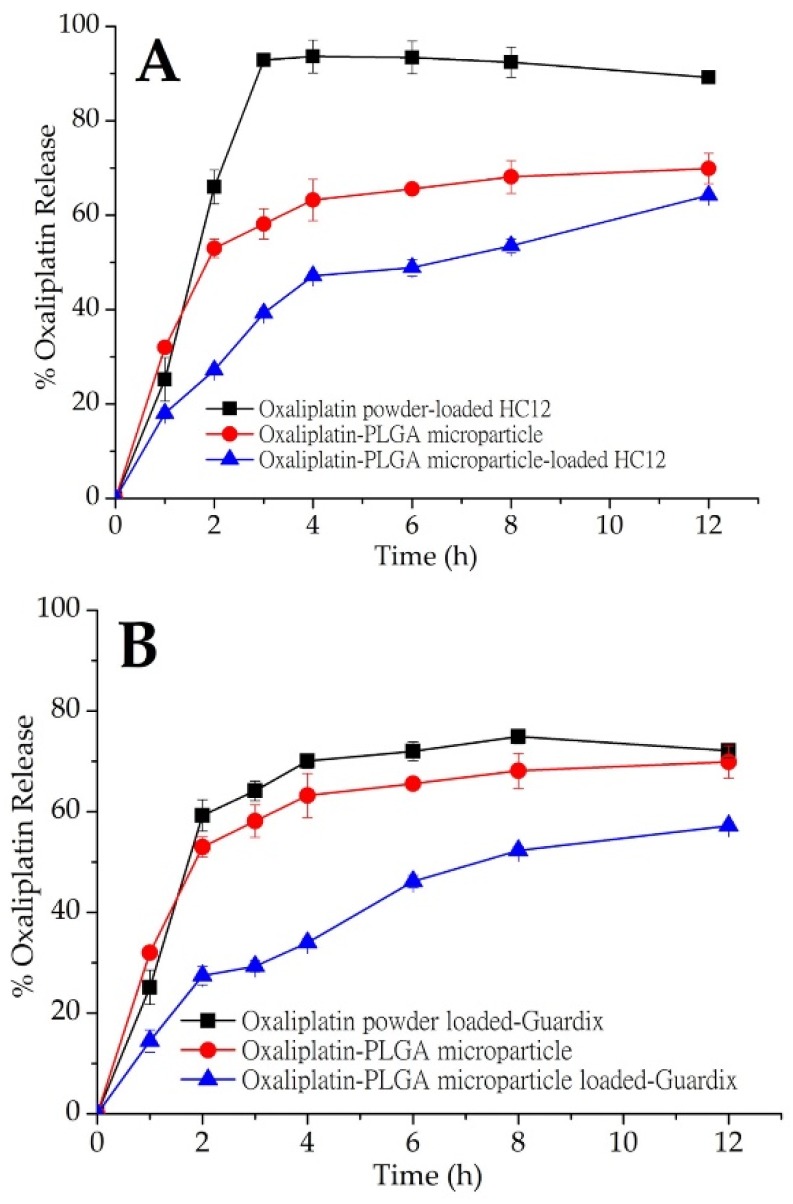

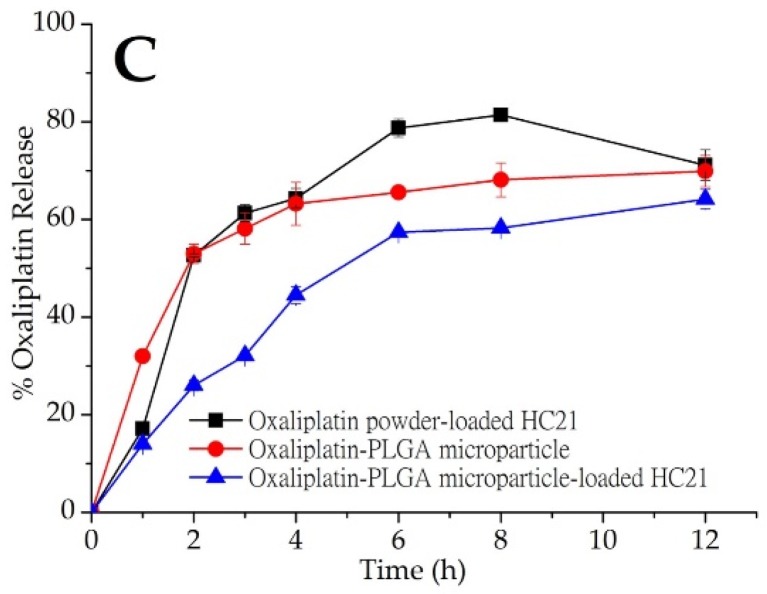
In vitro oxaliplatin release from PLGA microparticles (red, ●). Oxaliplatin release profile from oxaliplatin powder and oxaliplatin-PLGA microparticles-loaded in (**A**) HC12; (**B**) Guardix-Sol^®^; and (**C**) HC21 hydrogel. Mean ± S.D. (n = 3).

An in vitro oxaliplatin release study was conducted on oxaliplatin-PLGA microparticles generated using the double emulsion method. In addition, oxaliplatin-PLGA microparticles and oxaliplatin powder were dispersed into hydrogels (HC12, HC21, and Guardix-Sol^®^), and an in vitro oxaliplatin release study was conducted. Plots of cumulative oxaliplatin release (%) versus time (h) are shown in Figure 3A–C. In vitro release profile for oxaliplatin-PLGA microparticles (red closed circle; (●) showed immediate release. With an initial burst release, about 63.2% oxaliplatin was released at 4 h. Oxaliplatin release from PLGA microparticles is a combination of diffusion and bioerosion mechanism [26]. During the diffusion stage, oxaliplatin release occurs by diffusion through aqueous channels. The release could also be triggered by the external pores on the surface or through leaching of the oxaliplatin at or near the surface, leading to an initial burst release. Besides, the standard deviation values of the results of the in vitro release study for the hydrogels were low, which indicate excellent reproducibility of the release behavior. However, when oxaliplatin-PLGA microparticles were loaded into three different hydrogel systems, HC12, HC21, and Guardix-Sol^®^, slight initial burst release was observed. Although the rate of burst release was almost the same for the three systems, HC12, with the lowest viscosity, showed the maximum release. In contrast, the higher the viscosity of the hydrogel system, the lower the burst and sustained release observed. This may be due to low leaching of the drugs and high protection of the microparticles by the hydrogels.

Table 2 demonstrated the kinetic models adopted for evaluation. Where M_t_ was the cumulative drug released at time t and M was the initial drug present in the PLGA microparticles-loaded hydrogels. k_1_, k_H_, and k_KP_ are the first order, Higuchi, and Korsmeyer-Peppas release constant, respectively. M_t_/M_∞_ was the fraction of drug released at time t, and n was the diffusional release exponent symbolic of the release mechanism. The rate of oxaliplatin release from the HC12, Guardix-Sol^®^ and HC21-loaded PLGA microparticles were slower and constant compared with the oxaliplatin powder-loaded hydrogels, as indicated by the K_H_ values of 18.9686, 17.5626, and 20.6559% h^1/2^, respectively. Release constants were determined using the slope of the appropriate plots, and the regression coefficient (R^2^) was obtained through linear regression analysis (Table 2). The coefficient of determination (R^2^) was used as an indicator of curve fit for each of the considered models [27]. The regression coefficients R^2^, from the Korsmeyer-Peppas plots were 0.9342, 0.9554, and 0.9366 for HC12, Guardix-Sol^®^ and HC21, respectively, thus the log of cumulative drug release was proportional to the log of time. The best linear fits were observed for the PLGA microparticles-loaded hydrogels using both the Higuchi and Korsmeyer-Peppas models, suggesting that oxaliplatin release from the PLGA microparticles-loaded hydrogels were diffusive process. Oxaliplatin gradually dissolved into the fluid within the swelled hydrogels, then slowly diffused from polymeric networks of the hydrogels.

Although hydrogels are swellable materials with high water content, which readily allow the release or leaching of hydrophilic drugs through the channels, high viscosity might hinder this process. Therefore, release may be completed through bioerosion. In this study, three different hydrogel systems—HC12, HC21, and Guardix-Sol^®^, were loaded with oxaliplatin powder and oxaliplatin-PLGA microparticles. The HC12 hydrogel system (low viscosity) was loaded with oxaliplatin powder and more than 90% oxaliplatin release occurred within 3 h, followed by saturated release behavior up to 12 h. However, oxaliplatin-PLGA microparticles loaded into the HC12 hydrogel showed a slower release pattern than that of the oxaliplatin powder. Approximately 50% oxaliplatin was released after 4 h, followed by 64.2% release up to the end of the experiment (*p* < 0.05). Due to the less hydrophilicity of the PLGA copolymers, they absorb less water, and subsequently degrade more slowly. Therefore, drug release from the PLGA microparticles was delayed. In the case of Guardix-Sol^®^, with moderate viscosity, a sustained release profile was observed for the oxaliplatin-PLGA microparticles. During the first 2 h of the release study, less than 30% of oxaliplatin was released; however, 59.25% of oxaliplatin was released after the same amount of time when loaded with oxaliplatin powder (*p* < 0.05).

In addition, the HC21 hydrogel system (high viscosity) showed a similar sustained release profile when loaded with oxaliplatin-PLGA microparticles. In all three systems, more rapid oxaliplatin release was observed when oxaliplatin was loaded as a powder compared to that when loaded as microparticles. The release rate was faster than those of both oxaliplatin-PLGA microparticles and oxaliplatin-PLGA microparticles-loaded hydrogels. Additionally, both Guardix-Sol^®^ and HC21 showed similar sustained release patterns up to 12 h. Although PLGA microparticles were generated using the well-known double emulsion method, further studies are necessary to establish process parameters and extensive characterization of the particles.

### 3.4. Pharmacokinetics in Rats

**Figure 4:**
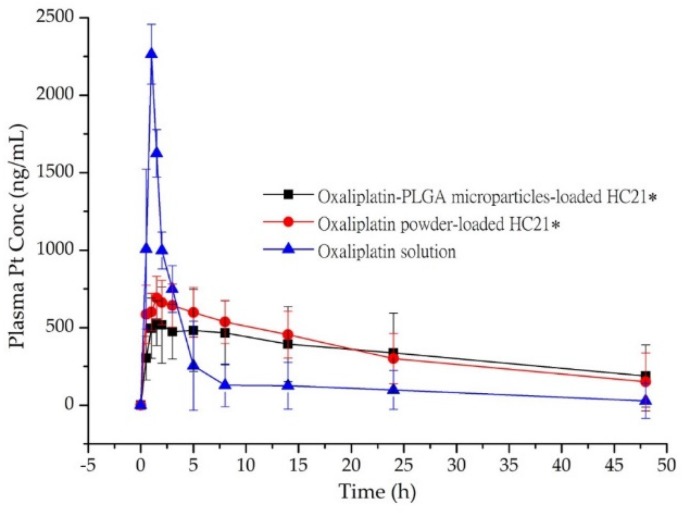
Plasma concentration-time profile of Pt in rats after intra-peritoneal administration of oxaliplatin powder and oxaliplatin-PLGA microparticles-loaded in HC21 hydrogel, and oxaliplatin solution. Data are expressed as the Mean ± standard deviation (n = 5). *, *p* < 0.05 represents a significant difference, two-sided RM ANOVA and Bonferroni test.

The bioavailability of oxaliplatin-PLGA microparticles loaded into the HC21 hydrogel was evaluated in rats. Figure 4 shows the mean concentration–time profiles of oxaliplatin in rats after a single dose (5 mg/kg) of oxaliplatin solution, oxaliplatin-PLGA microparticles, and oxaliplatin powder loaded into the HC21 hydrogel. The samples were introduced through a midline incision to open the peritoneum immediately after introducing intra-abdominal adhesions by multiple scraping to mimic postoperative surgical conditions. Intraperitoneal absorption of oxaliplatin solution was obviously higher than that of the hydrogel loaded samples (oxaliplatin-PLGA microparticles and oxaliplatin powder). For oxaliplatin solution AUC_0–48h_, C_max_, and T_max_ were 8181.51 ± 176.89 ng·h/mL, 2265.28 ± 192.51 ng/mL, and 1.0 h, respectively. In the case of oxaliplatin powder loaded into the HC21 hydrogel, AUC_0–48h_, C_max_, and T_max_ were 16,571.37 ± 139.13 ng·h/mL, 690.63 ± 140.54 ng/mL, and 1.5 h, respectively. Intraperitoneal bioavailability of oxaliplatin was significantly increased by up to 2-fold in the HC21 hydrogel (Table 3). In particular, 1.9-fold higher bioavailability was observed in the case of oxaliplatin-PLGA microparticles loaded into the HC21 hydrogel compared to that of the oxaliplatin solution. AUC_0–48h_, C_max_, and T_max_ were 16,012.12 ± 188.75 ng·h/mL, 528.75 ± 144.50 ng/mL, and 1.5 h, respectively. Moreover, mean residence time (MRT) was increased by 2.7-fold for oxaliplatin-PLGA microparticles when loaded into the HC21 hydrogels (*p* < 0.05). When the oxaliplatin-PLGA microparticles were loaded into the HC21 hydrogel, oxaliplatin release rate decreased due to the multi-layered encapsulation, which was observed from the in vitro release study. Therefore, in the high, moderate, and low viscous hydrogels (HC21, Guardix-Sol^®^, and HC12, respectively), oxaliplatin release from the PLGA microparticles, followed by release from the hydrogels, was observed in a sustained manner (Figure 3). Furthermore, using sustained-release hydrogels allows a longer residence time of the component at the application site (intraperitoneal after cytoreductive surgery), which offers additional benefits through the prevention of intra-abdominal adhesion and improvement in the delivery of intraperitoneal oxaliplatin from the PLGA microparticles.

### 3.5. Intraperitoneal Anti-Adhesion Effect

**Figure 5:**
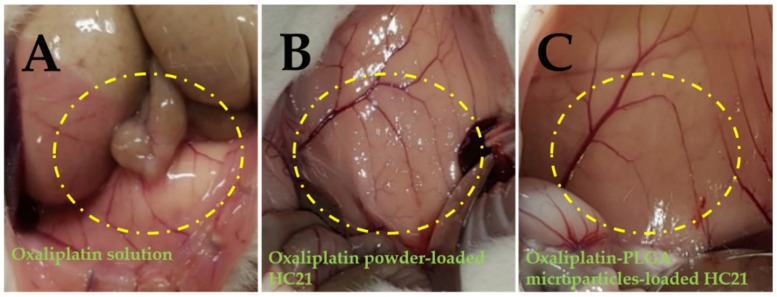
Photographical expression of in vivo anti-adhesion efficacy observed in rats after introducing intra-peritoneal injury. (**A**) oxaliplatin solution; (**B**) oxaliplatin powder-loaded HC21 hydrogel; and (**C**) oxaliplatin-PLGA microparticles-loaded HC21 hydrogel.

An extensive investigation of the intraperitoneal anti-adhesion efficacy of HC hydrogels was conducted in our previous study, after introducing peritoneal injury to generate peritoneal adhesion [18]. HC hydrogel groups (treated) showed the highest anti-adhesion barrier compared to the control group (non-treated). In the present study, the pharmacokinetic parameters of the PLGA microparticles loaded into the HC21 hydrogel were evaluated and compared with those of oxaliplatin powder loaded into hydrogels. Artificial injury on the left abdominal sidewall was introduced to mimic surgical conditions and observe the anti-adhesion barrier effect of hydrogel samples followed by the intraperitoneal delivery of oxaliplatin. The method was adopted from experienced surgeons certainly feasible in human administration after cytoreductive surgery in colorectal cancer treatment. Intraperitoneal anti-adhesion effect was observed and recorded photographically. Figure 5 shows a photographical representation of adhesion characteristics of the study groups.

HC21 hydrogel samples exhibited an efficient anti-adhesion barrier effect towards the injury. Although hydrogels were not intended to cure the injury, long resident time hydrogels significantly prevented adhesion along with rapid recovery of the injury, possibly due to mechanical separation from the injury site [28]. As reported previously, the washing away or displacement of the oxaliplatin solution from the application site allows less barrier or protection to the injury site [29]. Therefore, dense adhesion on the abdominal wall was observed, and, although current study was not intended to be carried out extensively, adhesion scoring and extent (as disclosed in a previous report) and photographic representation demonstrated the superiority of the hydrogels in terms of the anti-adhesion barrier. In the hydrogel-loaded samples, no or minor adhesion was observed in the treated rat abdomen (Figure 5B,C). Although two different samples were evaluated after loaded in HC21 hydrogel both oxaliplatin powder and oxaliplatin-PLGA microparticles loaded hydrogels showed significant improvement as anti-adhesion barrier. Subsequently, both hydrogel systems showed rapid wound recovery effect may be due to mechanical separation- could be seen from the blood spot in Figure 5C. Instead, oxaliplatin solution presents no barrier to the injure site and dense abdominal adhesion was observed. The HC21 hydrogel-loaded sample showed enhanced anti-adhesion protection to the injury site, suggesting its potential therapeutic application in CRC patients who have undergone cytoreductive surgery. Although the current study evaluated the pharmacokinetic profile of PLGA microparticle-loaded HC hydrogels, a further pharmacodynamics study on a CRC model is required to evaluate the composition of the hydrogels.

## 4. Conclusions

Oxaliplatin-PLGA microparticles were loaded into hydrogels to improve intraperitoneal chemotherapy with an anti-adhesion barrier effect. In an in vitro release study, we observed sustained oxaliplatin release from PLGA microparticles loaded into hydrogels in comparison with the unloaded sample. Moreover, in the in vivo pharmacokinetics study on rats, PLGA microparticle-loaded hydrogels showed higher bioavailability compared to oxaliplatin solution. Furthermore, visual observations revealed that the HC hydrogels were effective as an intra-abdominal anti-adhesion barrier. Therefore, intraperitoneal delivery of PLGA microparticle-loaded hydrogels, which have an effective intra-abdominal anti-adhesion barrier, is expected to provide an optimized alternative therapy for CRC. Earlier reports of cytotoxicity study for PLGA (RG502H) microparticles exhibited non-cytotoxic at increasing concentration [30]. Additionally, both HA and CMCNa are considered as inert and non-cytotoxic. However, cytotoxicity profile for the novel oxaliplatin-PLGA microparticles-loaded HC hydrogels were yet to revealed. As the investigation undertaken in current study was preliminary, pharmacokinetic and anti-adhesion efficacy of this multi-complex drug delivery system, further studies are required to address cytotoxicity, cell viability, toxicity in liver function, and efficacy in CRC model in vivo.

## Figures and Tables

**Table 1:** Compositions and characterizations of Poly-(d,l-lactide-*co*-glycolide) (PLGA) microparticles and hyaluronic acid (HA) and carboxymethyl cellulose sodium (CMCNa)-based cross-linked (HC) hydrogels.

Particles	Method	Weight Ratio				Particle Size (nm) (Mean ± SD)	Encapsulation Efficiency (%) (Mean ± SD)
		Oxaliplatin	PLGA 502 H	HA	CMCNa		
Oxaliplatin-PLGA Microparticles	Double Emulsion	1	50	-	-	1100.4 ± 257.7	77.9 ± 2.8
HC12 Hydrogel	Synthesis by cross-linking reaction	-	-	1	2	-	-
HC21 Hydrogel		-	-	2	1	-	-

**Table 2:** Release rate constants and regression coefficient R^2^ obtained from drug release profile based on kinetic equations.

Equations	Loaded in HC12 Hydrogel		Loaded in Guardix-Sol^®^ Hydrogel		Loaded in HC21 Hydrogel		PLGA Microparticle
	Oxaliplatin Powder	PLGA Microparticle	Oxaliplatin Powder	PLGA Microparticle	Oxaliplatin Powder	PLGA Microparticle	
^a^ Higuchi model: k_H_ (% h^1/2^)	28.5970	18.9686	22.1635	17.5626	24.3401	20.6559	20.0882
R^2^	0.6738	0.9611	0.7431	0.9783	0.7746	0.9485	0.8026
^b^ First—Order model: k_1_ (h^−1^)	0.0302	0.0421	0.0263	0.0469	0.0379	0.0522	0.0219
R^2^	0.1639	0.6646	0.2327	0.7224	0.2640	0.6451	0.4145
^c^ Korsmeyer—Peppas model: k_KP_	0.4517	0.5033	0.3750	0.5475	0.5352	0.6308	0.2884
R^2^	0.5495	0.9342	0.6099	0.9554	0.6512	0.9366	0.7767

^a^ Higuchi model: (Mt = k_H_ × t^1/2^); ^b^ First—Order model: (ln M_t_ = lnM + k_1_ × t); ^c^ Korsmeyer—Peppas model: (Mt/M∞ = k_KP_ × t^n^).

**Table 3:** In vivo pharmacokinetic parameters after intra-peritoneal administration to rats (n = 5).

Formulations	Pharmacokinetic Parameters			
	C_max_ (ng/mL)	T_max_ (h)	AUC_0–48h_ (ng·h/mL)	MRT (h)
Oxaliplatin solution	2265.28 ± 192.51	1	8181.51 ± 176.89	4.36 ± 0.65
Oxaliplatin powder loaded HC21	690.63 ± 140.54	1.5	16571.37 ± 139.13	10.29 ± 0.33
Oxaliplatin-PLGA microparticle loaded HC21	528.75 ± 144.50	1.5	16012.12 ± 188.75	12.02 ± 0.41
